# Prehospital Resuscitative Thoracotomy for Traumatic Cardiac Arrest

**DOI:** 10.1001/jamasurg.2024.7245

**Published:** 2025-02-26

**Authors:** Zane B. Perkins, Robert Greenhalgh, Ewoud ter Avest, Shadman Aziz, Andrew Whitehouse, Steve Read, Liz Foster, Frank Chege, Christine Henry, Richard Carden, Laura Kocierz, Gareth Davies, Tom Hurst, Robbie Lendrum, Stephen H. Thomas, David J. Lockey, Michael D. Christian

**Affiliations:** 1 London’s Air Ambulance, London, United Kingdom; 2 Centre for Trauma Sciences, Queen Mary University of London, London, United Kingdom; 3 Barts Health NHS Trust, London, United Kingdom; 4 University Medical Centre Groningen, University of Groningen, Groningen, the Netherlands; 5 London Ambulance Service NHS Trust, London, United Kingdom; 6 Manx Care, Nobles Hospital, Douglas, Isle of Man; 7 Department of Emergency Medicine, Harvard Medical School and Beth Israel Deaconess Medical Center, Boston, Massachusetts; 8 University of British Columbia, Vancouver, British Columbia, Canada

## Abstract

**Importance:**

Traumatic cardiac arrest (TCA) presents a critical challenge in trauma care, often occurring rapidly after injury before effective interventions are available.

**Objective:**

To evaluate the association of prehospital resuscitative thoracotomy with survival outcomes for TCA.

**Design, Setting, and Participants:**

This retrospective cohort study examined all cases of prehospital resuscitative thoracotomy for TCA in London from January 1999 to December 2019. Data were analyzed from July 2022 to July 2023.

**Exposure:**

Prehospital resuscitative thoracotomy for TCA.

**Main Outcomes and Measures:**

The primary outcome was survival to hospital discharge. Secondary outcomes included survival to hospital admission and neurological status at discharge.

**Results:**

Prehospital resuscitative thoracotomy was undertaken in 601 patients with out-of-hospital TCA. The median (IQR) age was 25 (20-37) years; 538 (89.5%) were male and 63 (10.5%) female. A total of 529 patients (88.0%) had a penetrating mechanism of injury. TCA occurred at a median (IQR) of 12 (6-22) minutes after the emergency call, with 491 arrests (81.7%) before the advanced trauma team’s arrival. TCA was the result of cardiac tamponade (105 patients, 17.5%), exsanguination (418 patients, 69.6%), and exsanguination combined with cardiac tamponade (72 patients, 12.0%). Thirty patients (5.0%) survived to hospital discharge, with a favorable neurological outcome observed in 23 survivors (76.6%). Survival varied significantly with the cause of TCA: 22 of 105 patients (21%) with cardiac tamponade, 8 of 418 patients (1.9%) with exsanguination, and none of the 72 patients with combined or other pathologies survived. There were no survivors beyond 15 minutes of TCA for cardiac tamponade and 5 minutes after exsanguination. Multivariable analysis revealed that the cause of TCA (adjusted odds ratio [aOR], 21.1; 95% CI, 8.1-54.7; *P* < .001), duration of TCA (aOR, 20.9; 95% CI, 4.4-100.6, *P* < .001), and absence of the need for internal cardiac massage (AOR, 0.2; 95% CI, 0.06-0.5; *P* = .001) were independently associated with survival.

**Conclusions and Relevance:**

TCA occurs soon after injury, with only a brief window available for effective intervention. This study found that resuscitative thoracotomy is feasible in a mature, physician-led, urban prehospital system and is associated with improved survival for patients with out-of-hospital TCA, particularly when caused by cardiac tamponade, in situations where other treatment options are limited.

## Key Points

**Question:**

Is prehospital resuscitative thoracotomy associated with improved survival rates for patients with traumatic cardiac arrest (TCA)?

**Findings:**

This cohort study found that prehospital resuscitative thoracotomy was associated with significantly improved survival in patients with TCA due to cardiac tamponade when performed within 10 minutes of arrest. Resuscitative thoracotomy was less effective for exsanguination-induced TCA.

**Meaning:**

Resuscitative thoracotomy is a feasible intervention for TCA in a mature, physician-led, urban prehospital system when performed soon after injury.

## Teaser

This cohort study examines more than 20 years of data for patients with traumatic cardiac arrest to evaluate the association of prehospital resuscitative thoracotomy with survival outcomes.

## Introduction

Despite substantial advances in trauma care, the prognosis for patients who experience out-of-hospital traumatic cardiac arrest (TCA) remains poor.^1^ TCA, characterized by an inability to sustain spontaneous cardiac output, frequently arises from reversible causes such as exsanguinating hemorrhage, cardiac tamponade, or tension pneumothorax. The success of resuscitation depends on the rapid correction of these causes.^2^ However, the opportunity to intervene is often missed, as TCA typically occurs prehospital, where effective treatments are not normally available.

Resuscitative thoracotomy (RT) is an immediate, lifesaving intervention for TCA.^3^ Its primary goals are alleviating tamponade, restoring coronary perfusion, and controlling noncompressible exsanguinating hemorrhage. RT has been in use for over a century and remains the definitive intervention for cardiac tamponade as well as a key tactic in managing torso exsanguination.^4,5^ Within this context, the time between injury and intervention is the single most important determinant of survival.^6,7,8,9^ As such, trauma centers have streamlined their “door to intervention” times, often initiating the procedure immediately in the emergency department, sacrificing optimal surgical conditions to achieve optimal results.^3^ Similarly, some prehospital care systems, realizing the limited effectiveness of standard on-scene resuscitation for TCA, have prioritized rapid transport to the hospital.^10,11^ While this “scoop and run” strategy may reduce the incidence of out-of-hospital TCA, it offers minimal benefit to patients who have TCA before reaching hospital because TCA is almost always fatal without immediate intervention.^12,13^

The true prevalence of prehospital TCA and its subsequent impact on trauma outcomes is unclear. The trauma outcome literature’s overreliance on hospital-level data, which usually excludes prehospital deaths, introduces a significant survival bias.^9,13,14,15^ Population-level statistics reveal a grim reality: more than two-thirds of trauma fatalities occur before hospital arrival.^16^ This is amplified in rapidly lethal conditions, such as cardiac tamponade, where prehospital mortality rates approach 90%, often from otherwise salvageable injuries.^7,8,9,14,15,17^ Despite considerable advances in trauma care, survival from these conditions remains dismal and has not seen any appreciable improvement over time.^14,15^ This reflects the relative lack of progress in developing effective prehospital interventions for TCA compared with in-hospital care advances.^15,18^ To address this problem, London’s Air Ambulance (LAA) implemented prehospital RT into their service in the early 1990s to enable immediate treatment of patients with out-of-hospital TCA due to cardiac tamponade.^19,20^

The aim of this study was to describe the outcomes of patients in TCA who undergo RT in the prehospital setting. In addition, we aim to examine the relationship between TCA duration, underlying causes, and patient outcomes.

## Methods

### Study Design

This retrospective cohort study evaluated patient outcomes after prehospital RT by LAA from January 1, 1999, to December 31, 2019. Data were analyzed from July 2022 to July 2023. As per the institutional review, this study was classified as a service evaluation, waiving the need for full ethics committee oversight in alignment with UK Health Research Authority guidance. The study protocol was prospectively registered (UIN researchregistry6529) and received approval from the institutional Clinical Effectiveness Unit (registration No. 10445). It adheres to the Strengthening the Reporting of Observational Studies in Epidemiology (STROBE) guidelines for reporting observational studies.^21^

### Setting

The London Trauma System is an inclusive, regional network overseeing trauma care for Greater London, which has a predominantly urban population of nearly 10 million. It integrates the London Ambulance Service (LAS), LAA, 4 major trauma centers , and 22 trauma units. LAS coordinates prehospital care, with a dedicated paramedic in the control center screening approximately 5000 emergency calls daily and dispatching LAA to supplement the LAS response for major trauma cases. The LAA physician-paramedic team provides 24/7 advanced trauma care, attending approximately 2000 patients annually, one-third of whom have penetrating trauma. LAA’s primary aim is to deliver immediate lifesaving care while rapidly transferring seriously injured patients to one of London’s 4 major trauma centers. When necessary, the team can perform advanced on-scene interventions, including prehospital anesthesia, blood transfusion, resuscitative balloon occlusion of the aorta (REBOA), and RT. To manage high demand, LAA uses staggered shifts to ensure more than 1 team is available during peak times. The system also collaborates with neighboring emergency medical services (EMS) through mutual aid agreements for resilience.

### Study Population

We included all patients who underwent prehospital RT by the LAA team during the study period. Cases were excluded if RT was conducted within a licensed health care facility or by individuals other than on-duty LAA clinicians.

### Intervention

LAA’s management approach for penetrating trauma emphasizes minimizing on-scene time and interventions, while prioritizing rapid transfer to the nearest major trauma center. RT is only performed for patients with out-of-hospital TCA when clinically indicated. The indications evolved over the study period. Initially, RT was indicated after penetrating chest or epigastrium injuries when the duration of TCA was under 10 minutes.^22^ In 2012, the indications expanded to include penetrating injuries to other body regions for aortic compression and hemorrhage control and in select cases blunt trauma.^23^

The operative technique has remained consistent throughout the study period and has been described previously.^22,23^ It follows a rapid, stepwise approach using simple equipment: (1) bilateral finger thoracostomies in the fourth intercostal space; (2) if no immediate return of spontaneous circulation, a clamshell thoracotomy is performed in the same space; (3) a midline (inverted T) pericardiotomy to evacuate blood and clots; (4) inspection and repair of cardiac wounds; and (5) cardiac resuscitation, comprising 2-handed massage, manual aortic occlusion, and volume resuscitation. In 2012, prehospital red blood cell transfusion was introduced for volume resuscitation, with plasma transfusion added in 2018. RT training and clinical governance have remained rigorous and unchanged over the study period, involving one-on-one instruction, simulation, and practical surgical skills sessions. This intensive training regimen, combined with a rigorous case review process, ensures a consistent, high-quality approach across the service.

### Data Sources

Data on all LAA patients are prospectively collected, which includes the contemporaneous completion of a patient report form, electronic database, and the saving of patient monitor recordings. An LAA research nurse ensures daily data quality and completeness, supported by monthly audits to maintain data integrity. For thoracotomy cases, clinicians complete an additional narrative report that details the timing, indications, findings, and procedural details. Primary data are supplemented by additional sources, including the LAS computer-aided dispatch system records, patient medical records, imaging results, autopsy reports, police reports, and interviews with patients or their relatives. To confirm accuracy, timing data from patient monitors are cross-verified with those from the LAS computer-aided dispatch, with LAA patient monitor clocks synchronized to the LAS computer-aided dispatch system daily.

### Data Collection

Two clinical investigators independently extracted data for each patient using a standardized proforma.^24^ Discrepancies were resolved by consensus with a third independent reviewer. We adhered to the International Liaison Committee on Resuscitation consensus guideline for reporting out-of-hospital cardiac arrest, extracting the core data elements defined in the patient, process, and outcome domains.^25^ Two modifications were made: (1) the first monitored rhythm data point was modified to the monitored rhythm at time of the decision to perform thoracotomy, and (2) the targeted temperature management data point was not collected. Additional data on mechanism of injury, anatomical site of injury, prehospital fluid administration, prehospital blood transfusion, and technical aspects of the intervention were also collected. Four time points were recorded for each patient: the time of the initial emergency call, the time of cardiac arrest, the time the LAA team arrived with the patient, and the time of thoracotomy. To establish the pathophysiological cause of cardiac arrest, we relied on the LAA clinician’s procedural report, autopsy findings, and in-hospital surgical records.

### Outcomes

The primary outcome was survival to hospital discharge. Secondary outcomes included the rate of survival to hospital admission (survived event) and neurological outcome at hospital discharge. Survival to hospital admission was defined as a return of spontaneous circulation sustained until admission and transfer of care to medical staff at the receiving hospital. Neurological outcome at hospital discharge was assessed using the Cerebral Performance Categories score, where category 1 or 2 indicates favorable cerebral function, category 3 or 4 indicates unfavorable cerebral function, and category 5 indicates brain death.^25,26^

### Statistical Analysis

All statistical analyses were performed using Stata software, version 16.0 (StataCorp). Normality was assessed through q-norm plotting and the Shapiro-Wilk test. Non–normally distributed continuous data are reported as median (IQR), and categorical data as frequency and percentage. Participant characteristics were compared using Mann-Whitney-Wilcoxon, χ^2^, or Fisher exact tests as appropriate. Annual trends in continuous data were assessed using simple linear regression, while χ^2^ for trend was used for categorical data. Prognostic factors for survival to hospital discharge were identified using univariable and multivariable logistic regression, with all collected clinical variables tested in univariable analysis for inclusion in the multivariable model. Purposeful selection of variables with clinical relevance or a *P* value less than .25 was performed. No imputation was performed for missing data. The strength of association between risk factors and survival was quantified by unadjusted odds ratio (OR) and adjusted odds ratio (aOR) with 95% CIs. Model performance was assessed by discrimination using the area under the receiver operating characteristic curve, and calibration, using Hosmer-Lemeshow goodness-of-fit testing and calibration plots. A *P* value less than .05 was considered statistically significant.

## Results

Over the 21-year period, LAA attended 45 647 injured patients, of whom 3223 experienced TCA, with 601 (1.3%) undergoing prehospital RT. There was a significant annual increase in the number of trauma cases attended, the proportion of cases due to penetrating trauma (from 10.5% in 1999 to 31.6% in 2019; *P* < .001, χ^2^ test for trend), the number of TCA cases attended, and the number of RT procedures performed over the study period (eFigure in Supplement 1). The 601 patients who underwent RT are the focus of this study. Their median (IQR) age was 25 (20-37) years; 538 (89.5%) were male and 63 (10.5%) female. A total of 529 patients (88.0%) presented with penetrating trauma (Table 1).

**Table 1.** Baseline Characteristics of 601 Patients in TCA Who Underwent Prehospital Resuscitative Thoracotomy

Characteristic	Missing data, No. (%)	Overall (n = 601), No. (%)	Cause of TCA, No. (%)		
			Tamponade (n = 105)	Exsanguination (n = 418)	Tamponade and exsanguination (n = 72)
Age, median (range), y	4 (0.7)	25 (2-98)	25 (12-74)	25 (2-82)	25 (15-85)
Gender					
Male	0	538 (89.5)	101 (96.2)	367 (87.8)	64 (88.9)
Female		63 (10.5)	4 (3.8)	51 (12.2)	8 (11.1)
Mechanism of injury					
Penetrating	0	529 (88.0)	101 (96.2)	355 (84.9)	69 (95.8)
Nonballistic	0	442 (73.5)	93 (88.6)	294 (70.3)	53 (73.6)
Ballistic	0	87 (14.5)	8 (7.6)	61 (14.6)	16 (22.2)
Blunt	0	72 (12.0)	4 (3.8)	63 (15.1)	3 (4.2)
Road traffic collision					
Motorcyclist	0	22 (3.7)	1 (0.9)	18 (4.3)	1 (1.4)
Pedestrian	0	18 (3.0)	0	18 (4.3)	0
Motor vehicle	0	9 (1.5)	3 (2.9)	5 (1.2)	1 (1.4)
Cyclist	0	6 (1.0)	0	6 (1.4)	0
Fall from height	0	10 (1.7)	0	10 (2.4)	0
Other	0	7 (1.2)	0	6 (1.4)	1 (1.4)
Time intervals, median (IQR), min					
Emergency call to TCA	87 (14.5)	12 (6-22)	11 (5.5-20)	13 (7-24)	8 (4-15)
Emergency call to advanced trauma team arrival	0	20 (16-26)	20 (16.5-27)	20.5 (16-26)	20 (17-25)
Emergency call to thoracotomy	14 (2.3)	22 (17-29)	21 (17-31)	22 (17-30)	21 (17-26)
Transport time^a^	2 (1.2)	12 (8-18)	13 (8.5-16.5)	12 (8-18)	10 (8-19)
Arrest witnessed					
No	0	70 (11.6)	21 (20.0)	53 (12.7)	6 (8.3)
Yes	0	521 (86.7)	84 (80.0)	365 (87.3)	66 (91.7)
Public/police	0	248 (41.3)	35 (33.3)	173 (41.4)	37 (51.4)
Ambulance service	0	163 (27.1)	28 (26.7)	111 (26.6)	22 (30.6)
Advanced trauma team	0	110 (18.3)	21 (20.0)	81 (19.4)	7 (9.7)
Cardiac rhythm before thoracotomy	84 (14.0)				
Pulseless electrical activity					
Sinus rhythm		66 (12.8)	16 (18.6)	47 (13.2)	3 (4.4)
Sinus bradycardia		71 (13.7)	13 (15.1)	52 (14.6)	4 (5.9)
Agonal rhythm		61 (11.8)	8 (9.3)	44 (12.3)	7 (10.3)
Asystole		314 (60.7)	49 (57.0)	210 (58.8)	54 (79.4)
Ventricular fibrillation		1 (0.2)^b^	0	0	0
Resuscitation interventions before advanced trauma team arrival					
Supraglottic airway	4 (0.7)	146 (24.5)	23 (22.1)	109 (26.2)	13 (18.1)
Tracheal intubation^c^	4 (0.7)	197 (33.0)	36 (34.6)	125 (30.1)	34 (47.2)
Positive pressure ventilation	4 (0.7)	473 (79.2)	78 (75.0)	328 (78.8)	63 (87.5)
Pleural decompression^d^	4 (0.7)	71 (11.9)	12 (11.5)	44 (10.6)	15 (20.8)
External cardiac massage	4 (0.7)	451 (75.5)	77 (74.0)	307 (74.0)	64 (88.9)
Resuscitation interventions after advanced trauma team arrival					
Supraglottic airway	0	29 (4.8)	4 (3.8)	23 (5.5)	2 (2.8)
Tracheal intubation^c^	0	233 (38.8)	41 (39.1)	163 (39.0)	26 (36.1)
Tracheal intubation (drug assisted)	0	53 (8.8)	11 (10.5)	39 (9.3)	3 (4.2)
Positive pressure ventilation	0	601 (100)	105 (100)	418 (100)	72 (100)
Pleural decompression (thoracostomy)	0	601 (100)	105 (100)	418 (100)	72 (100)
Internal cardiac massage	12 (2.0)	513 (87.1)	92 (87.6)	347 (85.3)	68 (95.8)
Aortic occlusion	83 (13.8)	405 (78.2)	62 (73.8)	293 (80.1)	47 (74.6)
Defibrillation after thoracotomy	15 (2.5)	114 (19.5)	32 (31.7)	63 (15.5)	17 (23.6)
Resuscitation drugs administered					
Adrenaline	0	266 (44.3)	66 (62.9)	167 (40.0)	30 (41.7)
Calcium	0	153 (25.5)	31 (29.5)	112 (26.8)	9 (12.5)
Sodium bicarbonate	0	123 (20.5)	29 (27.6)	84 (20.1)	9 (12.5)
Resuscitation fluids administered	0	548 (91.2)	102 (97.1)	376 (90.0)	65 (90.3)
Crystalloid/colloid	0	402 (66.9)	78 (74.3)	264 (63.2)	55 (76.4)
Prehospital blood transfusion	0	312 (51.9)	53 (50.5)	229 (54.8)	29 (40.3)

Abbreviation: TCA, traumatic cardiac arrest.

^a^ Leaving scene to arrival at hospital time for patients with a return of spontaneous circulation who were transported to hospital (n = 160).

^b^ Ventricular fibrillation as the presenting rhythm before thoracotomy occurred in a patient with blunt myocardial contusion.

^c^ Tracheal intubation without drugs.

^d^ Needle chest decompression or thoracostomy.

### Timelines

**Figure 1.**
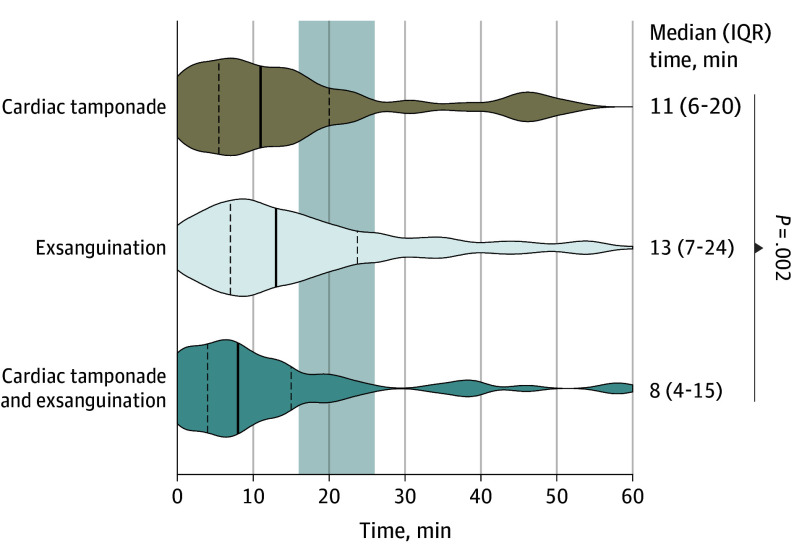
Violin Plot of the Time (in Minutes) From the Emergency Call to the Onset of Traumatic Cardiac Arrest (TCA) by Cause of TCA Vertical lines within the violin plots indicate the median (IQR). The shaded background indicates the median (IQR) time from the emergency call to arrival of the prehospital advance trauma team. Comparisons used a Mann-Whitney test.

From the emergency call, the median (IQR) time to reported TCA onset was 12 (6-22) minutes and to the advanced trauma team’s arrival was 20 (16-26) minutes (Figure 1). On arrival, 481 patients (80.0%) were already in established TCA. The median (IQR) time between the emergency call and thoracotomy was 22 minutes (17-29) minutes.

### Pathophysiological Cause of TCA

The underlying cause of TCA was documented in 597 of 601 cases (99.3%). Cardiac tamponade was confirmed in 105 patients (17.6%), exsanguination in 418 patients (70.0%), or a combination of both in 72 patients (12.1%). Only 2 cases had other causes: 1 major airway injury and 1 ventricular fibrillation due to myocardial contusion. The anatomical injuries precipitating TCA are reported in eTables 1 and 2 in Supplement 1.

### Factors Associated With Survival

In unadjusted analyses, survival was significantly associated with the cause of TCA (tamponade, 21.0%, vs other cause, 1.6%), duration of TCA (15.7% for <1 minute, 9.2% for 1-5 minutes, 2.6% for >5-10 minutes, 0.8% for >10 minutes), witnessed TCA (witnessed, 14.3%, vs unwitnessed, 2.8%), cardiac rhythm at the time of thoracotomy (pulseless electrical activity, 16.1%, vs asystole or an agonal rhythm, 1.8%), cardiopulmonary resuscitation before thoracotomy (yes, 2.4%, vs no, 13.0%), internal cardiac massage (internal massage, 3.5%, vs no massage, 15.8%), and epinephrine administration (epinephrine, 3.0%, vs no epinephrine, 6.6%). Multivariable analysis revealed that the cause of TCA (aOR, 21.1; 95% CI, 8.1-54.7; *P* < .001), duration of TCA (aOR, 20.9; 95% CI, 4.4-100.6, *P* < .001), and absence of the need for internal cardiac massage (aOR, 0.2; 95% CI, 0.06-0.5; *P* = .001) were independently associated with survival.(Table 2). The multivariable model had excellent discrimination (area under the curve, 0.93; 95% CI, 0.89-0.97) and calibration (Hosmer-Lemeshow goodness of fit *P* = .91).

**Table 2.** Multivariable Logistic-Regression Analysis of Clinical Factors Associated With Survival to Hospital Discharge

Independent variable^a^	Adjusted OR (95% CI)	*P* value
Cause of TCA (tamponade)	21.1 (8.1-54.7)	<.001
Duration of TCA, min		
<1	20.9 (4.4-100.6)	<.001
1-5	18.9 (3.5-101.0)	.001
>5-10	4.9 (0.8-32.2)	.10
>10	1 [Reference]	NA
Internal cardiac massage	0.2 (0.06-0.5)	.001

Abbreviations: NA, not applicable; OR, odds ratio; TCA, traumatic cardiac arrest.

^a^ All clinical variables (Table 1) were assessed for inclusion in the multivariable model. Variables were selected based on clinical relevance or *P* value <.25 in univariable analysis.

### Clinical Outcomes

Follow-up was complete for all patients. Overall, 30 patients (5.0%) survived to hospital discharge. The majority of survivors (23/30, 76.7%) had a favorable neurological outcome. Of the nonsurvivors, 160 patients (26.6%) survived the event while 441 (73.4%) were pronounced dead at the scene.

#### Cardiac Tamponade

**Figure 2.**
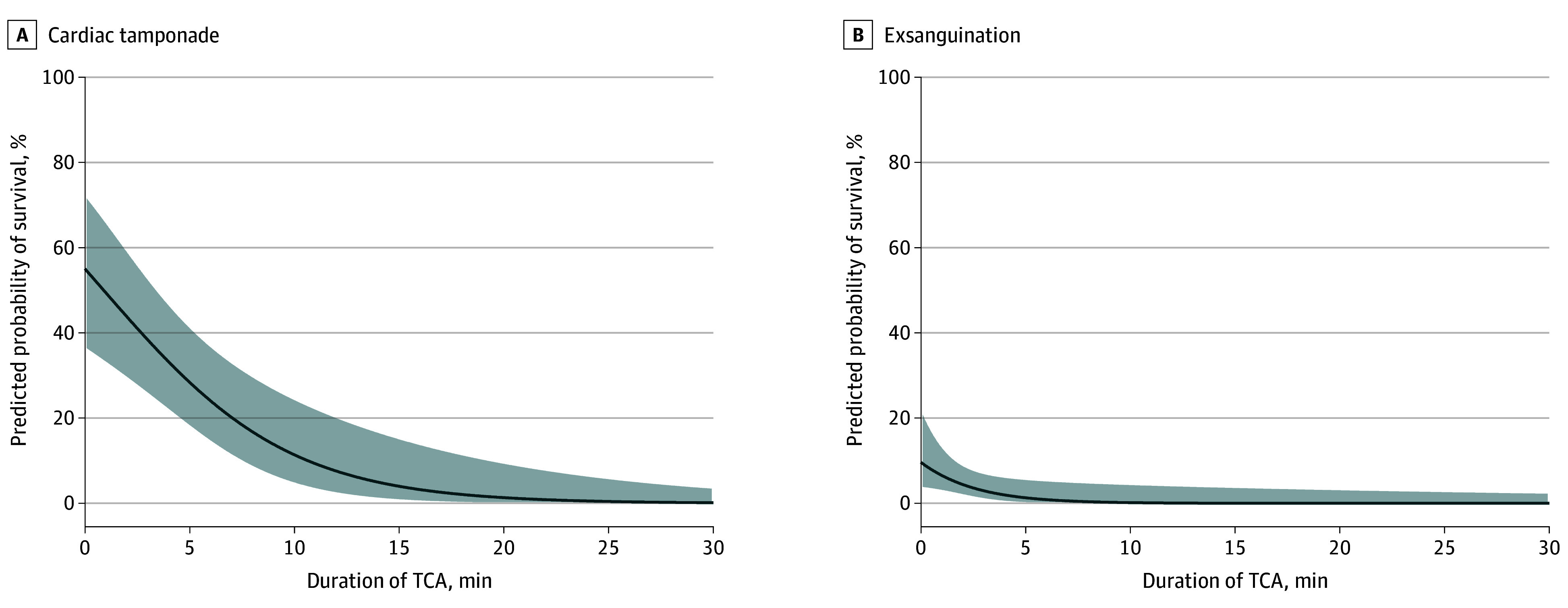
Predicted Probability of Survival After Traumatic Cardiac Arrest (TCA) Caused by (A) Cardiac Tamponade and (B) Exsanguination According to the Duration of TCA in Minutes The predicted probability of survival was calculated using simple logistic regression with the duration of TCA in minutes as the independent variable and a binary outcome of survived vs died as the dependent variable. The shading indicates the 95% asymptotic confidence bands of the true curve.

**Figure 3.**
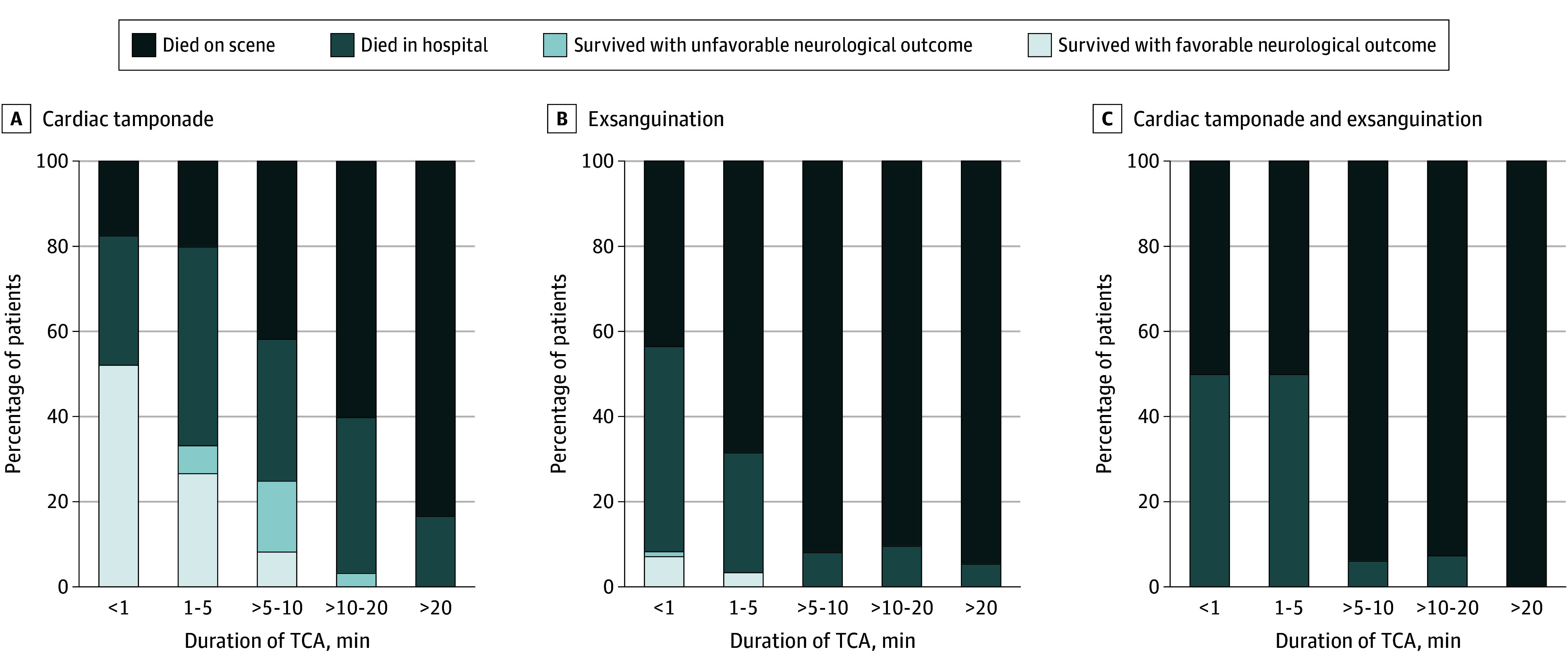
Outcomes for Traumatic Cardiac Arrest (TCA) Treated With Prehospital Resuscitative Thoracotomy According to the Duration of TCA Neurological outcome at hospital discharge was assessed using the Cerebral Performance Categories score, where category 1 or 2 indicates good neurological survival and category 3 or 4 indicates poor neurological survival.

Among the 105 patients in TCA due to cardiac tamponade, 22 (21.0%) survived to hospital discharge (eTable 3 in Supplement 1). The probability of survival decreased with each additional minute of TCA (Figure 2A). For the 92 patients (87.6%) with known duration of TCA, survival was 52.2% (12/23) when under 1 minute; 33.3% (5/15) when 1 to 5 minutes; 25.0% (3/12) when between 5 and 10 minutes; and 2.4% (1/42) when greater than 10 minutes (*P* < .001, χ^2^ test for trend) (Figure 3A). There were no survivors beyond a 15-minute duration of TCA. Survival rates were higher in cases where TCA was witnessed by the advanced trauma team compared with those who arrested before their arrival (48.0% [12/25 patients] vs 12.5% [10/80 patients]; OR, 6.46; 95% CI, 2.19-17.90; *P* < .001). Survival was also associated with the electrocardiogram rhythm at time of thoracotomy: 48.3% (14/29 patients) with pulseless electrical activity and 12.3% (7/57 patients) in asystole or an agonal rhythm survived (OR, 6.67; 95% CI, 2.18-18.16; *P* < .001). Among the 22 survivors, 16 (72.7%) had a favorable neurological outcome, with a median (IQR) TCA duration significantly shorter than those with unfavorable neurological outcome (1 [1-2] minutes vs 8 [5-13] minutes; *P* < .001). Among the nonsurvivors, 59 patients (55.7%) survived the event while 46 patients (43.8%) died at the scene. The relationship between patient outcomes and duration of TCA is shown in Figure 3A. No significant survival difference was observed between cardiac tamponade caused by penetrating or blunt trauma (20.8% [21/101 patients] vs 25% [1/4 patients]; *P* > .99)

#### Exsanguination

Among the 418 patients in TCA from exsanguination, 8 (1.9%) survived to hospital discharge (eTable 4 in Supplement 1). Survival was strongly associated with the duration of TCA, with no survivors beyond 5 minutes (Figure 2B). Survival rates were higher in cases where TCA was witnessed by the advanced trauma team compared with those who arrested before their arrival (6.9% [6/87 patients] vs 0.6% [2/331 patients]; OR, 12.2; 95% CI, 2.91-59.80; *P* = .001). Pulseless electrical activity was present at RT in 99 patients and all survivors (8/99 patients, 8.1%), while none with asystole or an agonal rhythm (0/258) survived. The majority of survivors (7/8 patients, 87.5%) had a favorable neurological outcome. Among the nonsurvivors, 82 patients (20.0%) survived the event, and 328 patients (80.0%) died at the scene. The relationship between patient outcomes and duration of TCA is shown in Figure 3B. Compared with cardiac tamponade, patients with TCA due to exsanguination experienced significantly worse outcomes (eTable 5 in Supplement 1). Blood product resuscitation did not significantly improved survival (2.2% [5/229] vs 1.6% [3/189]; OR, 1.38; 95%CI, 0.36-5.28; *P* = .73).

#### Cardiac Tamponade and Exsanguination

None of the 72 patients in TCA due to combined cardiac tamponade and exsanguination survived to hospital discharge. The onset of TCA occurred more rapidly after injury compared with cases with either pathology alone, with fewer events witnessed by the advance trauma team (Table 1 and Figure 1). Nine patients (12.5%) survived the event but died in the hospital, while 63 (87.5%) were pronounced dead at the scene (Figure 3C).

## Discussion

TCA often occurs soon after injury, with only a brief window available for effective intervention. Transfer to the hospital in established TCA is almost always futile.^3,4,12^ This study demonstrates that RT is feasible in the prehospital setting and offers a resuscitation option associated with improved survival for patients in TCA, particularly when it is caused by cardiac tamponade.

The onset of TCA typically occurs within 10 to 15 minutes postinjury, posing significant challenges for timely intervention. Even in advanced EMS systems, prehospital times are rarely less than 30 minutes,^1^ and transport of patients in established TCA to the hospital has a dismal prognosis.^3,4,12^ Initiatives to reduce prehospital time by immediate police transport of penetrating trauma patients (scoop and run) have also failed to improve survival.^27,28,29,30,31^ This highlights the need for effective on-scene interventions for potentially reversible causes of TCA.

Immediate surgery is the most effective treatment for penetrating cardiac injuries and cardiac tamponade.^4,6^ However, almost 90% of patients with these injuries die because they cannot reach a surgeon in time.^7,8,9,14,15,17^ Our study confirms that prehospital RT, by allowing earlier intervention, is an effective treatment for TCA caused by cardiac tamponade, enabling neurologically intact survival in a significant proportion of patients who would otherwise have not survived. However, success requires a system capable of rapid deployment of a highly trained medical team, supported by a robust supervision, training, and clinical governance program.^2,20,22^ Furthermore, effective integration with EMS and hospital trauma services is essential to ensure continuity of care from the prehospital to in-hospital phases.^32^

Prehospital RT does not appear to be effective for TCA due to exsanguination, with a survival rate of only 1.6% (8/490 patients). The few survivors all had organized electrocardiogram activity and received large-volume fluid resuscitation by prehospital standards, suggesting they were in a “low-flow” state that responded to aggressive fluid resuscitation and attempts at hemorrhage control. All survivors underwent temporary aortic occlusion, which may have contributed to their survival. Advancements in prehospital care, including the availability of larger volumes of blood products and less invasive methods of aortic occlusion, such as REBOA, may offer more effective treatment options for exsanguination TCA in the future.^33,34^

Our findings for prehospital RT align with existing recommendations for in-hospital RT.^12,35^ First, the focus should be on patients with penetrating thoracic trauma, especially when cardiac tamponade is suspected, as they have the highest chance of survival. Second, the time-critical nature of the intervention is paramount^36^; our data demonstrate that performing RT within minutes of cardiac arrest is essential for neurologically intact survival. Consistent with previous studies, we observed no survivors for TCA durations longer than 15 minutes for cardiac tamponade and beyond 5 minutes for exsanguination.^35,37,38^ While diagnostic tools such as ultrasound can assist in determining the underlying cause, our results suggest that any delay may reduce the effectiveness of RT, particularly when cardiac tamponade is suspected. However, as blunt trauma–related tamponade is rare, stronger evidence of tamponade may be needed before proceeding with RT in these cases.

While the mechanism of injury (blunt or penetrating) is often emphasized in RT literature, our study highlights the pathophysiological cause of arrest as the true determinant of outcome. Tamponade, though more common after penetrating trauma, showed similar outcomes regardless of the injury mechanism. Thus, using the mechanism of injury as a surrogate in guidelines may risk excluding the rare patient with blunt trauma tamponade from a lifesaving treatment.

### Strengths and Limitations

This study’s primary strength is its large cohort size, which significantly adds to the limited existing literature on prehospital RT and allows for reliable statistical analysis. Additionally, the comprehensive data collection from multiple sources, combined with rigorous training and a robust clinical governance program, enhances data accuracy, consistency, and completeness, thereby improving the reliability of the findings.

However, this study also has several limitations. Because of its retrospective design, we relied on data recorded by attending clinical teams during TCA, which led to some inevitable data gaps, although overall data completeness was high. The data were collected over a 21-year period, during which evolving treatment options, such as the introduction of blood products and REBOA in the prehospital setting, may have influenced patient selection and treatment outcomes, potentially affecting survival rates. Selection bias is inherent in the decision to perform RT because only patients meeting specific indications received the intervention. Attribution bias may also have influenced the determination of the cause of TCA, but this risk was minimized by relying on objective clinical records, including procedural reports, autopsy findings, and surgical records. The relatively small number of patients with gunshot wounds in our cohort is another limitation. Given that outcomes after thoracotomy for gunshot wounds are significantly worse than those from stab wounds,^3,8,12,36,38^ our findings should be cautiously applied to populations predominantly affected by gunshot wounds. Additionally, the small number of blunt trauma cases with cardiac tamponade limits the robustness of conclusions for this subgroup, warranting cautious interpretation. The study’s setting within a large urban prehospital system may also limit the generalizability of the findings to other settings with different resources and protocols. Outside a system with a high incidence of penetrating knife injuries, maintaining operator proficiency for RT may be challenging. Finally, the challenges associated with conducting randomized studies on this topic are well documented,^36^ though efforts are ongoing in the UK to examine this intervention through a prospective, nonrandomized study.^39^

## Conclusions

This study demonstrates that RT is a feasible intervention for out-of-hospital TCA and is associated with improved survival, particularly for cases caused by cardiac tamponade and when performed within minutes of arrest. The findings emphasize the importance of rapid identification and treatment of reversible causes of TCA and highlight the need for effective prehospital interventions to achieve this.

## Notes

Supplement 1. **eTable 1.** Surface location of injuries resulting in Traumatic Cardiac Arrest from Cardiac Tamponade and/or Exsanguination. **eTable 2.** Cardiac injuries resulting in Traumatic Cardiac Arrest from Cardiac Tamponade and/or Exsanguination. **eTable 3.** Survivors of traumatic cardiac arrest from cardiac tamponade. **eTable 4.** Survivors of traumatic cardiac arrest from exsanguination. **eTable 5.** Outcomes of 523 patients in traumatic cardiac arrest caused by either cardiac tamponade or exsanguination. **eFigure.** Number of (A) cases attended, (B) Traumatic Cardiac Arrest cases attended and (C) pre-hospital Resuscitative Thoracotomy procedures performed per year over the study period.

Supplement 2. Data sharing statement

## References

[1] Millin MG, Galvagno SM, Khandker SR, Malki A, Bulger EM; Standards and Clinical Practice Committee of the National Association of EMS Physicians (NAEMSP); Subcommittee on Emergency Services–Prehospital of the American College of Surgeons’ Committee on Trauma (ACSCOT). Withholding and termination of resuscitation of adult cardiopulmonary arrest secondary to trauma: resource document to the joint NAEMSP-ACSCOT position statements. J Trauma Acute Care Surg. 2013;75(3):459-467. doi:10.1097/TA.0b013e31829cfaea 24089117

[2] Lott C, Truhlář A, Alfonzo A, ; ERC Special Circumstances Writing Group Collaborators. European Resuscitation Council Guidelines 2021: cardiac arrest in special circumstances. Resuscitation. 2021;161:152-219. doi:10.1016/j.resuscitation.2021.02.011 33773826

[3] Rhee PM, Acosta J, Bridgeman A, Wang D, Jordan M, Rich N. Survival after emergency department thoracotomy: review of published data from the past 25 years. J Am Coll Surg. 2000;190(3):288-298. doi:10.1016/S1072-7515(99)00233-1 10703853

[4] Cothren CC, Moore EE. Emergency department thoracotomy for the critically injured patient: objectives, indications, and outcomes. World J Emerg Surg. 2006;1(1):4. doi:10.1186/1749-7922-1-4 16759407 PMC1459269

[5] Pust GD, Namias N. Resuscitative thoracotomy. Int J Surg. 2016;33(Pt B):202-208. doi:10.1016/j.ijsu.2016.04.006 27102328

[6] Boyd TF, Strieder JW. Immediate surgery for traumatic heart disease. J Thorac Cardiovasc Surg. 1965;50(3):305-315. doi:10.1016/S0022-5223(19)33188-5 14346540

[7] Campbell NC, Thomson SR, Muckart DJ, Meumann CM, Van Middelkoop I, Botha JB. Review of 1198 cases of penetrating cardiac trauma. Br J Surg. 1997;84(12):1737-1740. 9448629

[8] Sugg WL, Rea WJ, Ecker RR, Webb WR, Rose EF, Shaw RR. Penetrating wounds of the heart: an analysis of 459 cases. J Thorac Cardiovasc Surg. 1968;56(4):531-545. doi:10.1016/S0022-5223(19)42812-2 5683690

[9] Kulshrestha P, Iyer K, Sampath KA, Sharma ML, Rao IM, Venugopal P. Cardiac injuries: a clinical and autopsy profile. J Trauma Acute Care Surg. 1990;30(2):203-207. doi:10.1097/00005373-199002000-00012 2304116

[10] Gervin AS, Fischer RP. The importance of prompt transport of salvage of patients with penetrating heart wounds. J Trauma. 1982;22(6):443-448. doi:10.1097/00005373-198206000-00001 7086909

[11] Seamon MJ, Fisher CA, Gaughan J, . Prehospital procedures before emergency department thoracotomy: “scoop and run” saves lives. J Trauma. 2007;63(1):113-120. doi:10.1097/TA.0b013e31806842a1 17622878

[12] Seamon MJ, Haut ER, Van Arendonk K, . An evidence-based approach to patient selection for emergency department thoracotomy: a practice management guideline from the Eastern Association for the Surgery of Trauma. J Trauma Acute Care Surg. 2015;79(1):159-173. doi:10.1097/TA.0000000000000648 26091330

[13] Vianen NJ, Van Lieshout EMM, Maissan IM, . Prehospital traumatic cardiac arrest: a systematic review and meta-analysis. Eur J Trauma Emerg Surg. 2022;48(4):3357-3372. doi:10.1007/s00068-022-01941-y 35333932 PMC9360068

[14] Rhee PM, Foy H, Kaufmann C, . Penetrating cardiac injuries: a population-based study. J Trauma. 1998;45(2):366-370. doi:10.1097/00005373-199808000-00028 9715197

[15] McNicoll CF, McNickle AG, Vanderet D, . Shot through the heart: a 17-year analysis of pre-hospital and hospital deaths from penetrating cardiac injuries. Injury. 2023;54(5):1349-1355. doi:10.1016/j.injury.2023.01.046 36764901

[16] Evans JA, van Wessem KJ, McDougall D, Lee KA, Lyons T, Balogh ZJ. Epidemiology of traumatic deaths: comprehensive population-based assessment. World J Surg. 2010;34(1):158-163. doi:10.1007/s00268-009-0266-1 19882185

[17] Demetriades D, van der Veen BW. Penetrating injuries of the heart: experience over two years in South Africa. J Trauma. 1983;23(12):1034-1041. doi:10.1097/00005373-198312000-00003 6655748

[18] Kang N, Hsee L, Rizoli S, Alison P. Penetrating cardiac injury: overcoming the limits set by Nature. Injury. 2009;40(9):919-927. doi:10.1016/j.injury.2008.12.008 19442973

[19] Keogh SP, Wilson AW. Survival following pre-hospital arrest with on-scene thoracotomy for a stabbed heart. Injury. 1996;27(7):525-527. doi:10.1016/0020-1383(96)00023-X 8977847

[20] Davies GE, Lockey DJ. Thirteen survivors of prehospital thoracotomy for penetrating trauma: a prehospital physician-performed resuscitation procedure that can yield good results. J Trauma. 2011;70(5):E75-E78. doi:10.1097/TA.0b013e3181f6f72f 21131854

[21] von Elm E, Altman DG, Egger M, Pocock SJ, Gøtzsche PC, Vandenbroucke JP; STROBE Initiative. The Strengthening the Reporting of Observational Studies in Epidemiology (STROBE) statement: guidelines for reporting observational studies. Lancet. 2007;370(9596):1453-1457. doi:10.1016/S0140-6736(07)61602-X 18064739

[22] Wise D, Davies G, Coats T, Lockey D, Hyde J, Good A. Emergency thoracotomy: “how to do it”. Emerg Med J. 2005;22(1):22-24. doi:10.1136/emj.2003.012963 15611536 PMC1726527

[23] Rehn M, Davies G, Lockey D. Resuscitative thoracotomy: a practical approach. Surgery (Oxf). 2018;36(8):424-428. doi:10.1016/j.mpsur.2018.04.009

[24] Harris PA, Taylor R, Thielke R, Payne J, Gonzalez N, Conde JG. Research electronic data capture (REDCap): a metadata-driven methodology and workflow process for providing translational research informatics support. J Biomed Inform. 2009;42(2):377-381. doi:10.1016/j.jbi.2008.08.010 18929686 PMC2700030

[25] Perkins GD, Jacobs IG, Nadkarni VM, ; Utstein Collaborators. Cardiac arrest and cardiopulmonary resuscitation outcome reports: update of the Utstein resuscitation registry templates for out-of-hospital cardiac arrest: a statement for healthcare professionals from a task force of the International liaison Committee on resuscitation (American heart association, European resuscitation Council, Australian and New Zealand Council on resuscitation, heart and stroke Foundation of Canada, InterAmerican heart Foundation, resuscitation Council of southern Africa, resuscitation Council of Asia); and the American heart association emergency cardiovascular care Committee and the Council on cardiopulmonary, critical care, perioperative and resuscitation. Circulation. 2015;132(13):1286-1300. doi:10.1161/CIR.0000000000000144 25391522

[26] Jennett B, Bond M. Assessment of outcome after severe brain damage. Lancet. 1975;1(7905):480-484. doi:10.1016/S0140-6736(75)92830-5 46957

[27] Band RA, Salhi RA, Holena DN, Powell E, Branas CC, Carr BG. Severity-adjusted mortality in trauma patients transported by police. Ann Emerg Med. 2014;63(5):608-614.e3. doi:10.1016/j.annemergmed.2013.11.008 24387925 PMC5912155

[28] Taghavi S, Maher Z, Goldberg AJ, . An analysis of police transport in an Eastern Association for the Surgery of Trauma multicenter trial examining prehospital procedures in penetrating trauma patients. J Trauma Acute Care Surg. 2022;93(2):265-272. doi:10.1097/TA.0000000000003563 35121705

[29] Maher Z, Beard JH, Dauer E, . Police transport of firearm-injured patients-more often and more injured. J Trauma Acute Care Surg. 2021;91(1):164-170. doi:10.1097/TA.0000000000003225 34108420

[30] Wandling MW, Nathens AB, Shapiro MB, Haut ER. Police transport versus ground EMS: a trauma system-level evaluation of prehospital care policies and their effect on clinical outcomes. J Trauma Acute Care Surg. 2016;81(5):931-935. doi:10.1097/TA.0000000000001228 27537514

[31] Winter E, Hynes AM, Shultz K, Holena DN, Malhotra NR, Cannon JW. Association of police transport with survival among patients with penetrating trauma in Philadelphia, Pennsylvania. JAMA Netw Open. 2021;4(1):e2034868-e2034868. doi:10.1001/jamanetworkopen.2020.34868 33492375 PMC7835719

[32] Konig T, Perkins Z, Davies G. Training non-surgeons to perform resuscitative thoracotomy. Scand J Trauma Resusc Emerg Med. 2013;21(suppl 1):A2. doi:10.1186/1757-7241-21-S1-A2

[33] Lendrum R, Perkins Z, Chana M, . Pre-hospital resuscitative endovascular balloon occlusion of the aorta (REBOA) for exsanguinating pelvic haemorrhage. Resuscitation. 2019;135:6-13. doi:10.1016/j.resuscitation.2018.12.018 30594600

[34] Pusateri AE, Moore EE, Moore HB, . Association of prehospital plasma transfusion with survival in trauma patients with hemorrhagic shock when transport times are longer than 20 minutes: a post hoc analysis of the PAMPer and COMBAT clinical trials. JAMA Surg. 2020;155(2):e195085-e195085. doi:10.1001/jamasurg.2019.5085 31851290 PMC6990948

[35] Burlew CC, Moore EE, Moore FA, . Western Trauma Association critical decisions in trauma: resuscitative thoracotomy. J Trauma Acute Care Surg. 2012;73(6):1359-1363. doi:10.1097/TA.0b013e318270d2df 23188227

[36] Liu A, Nguyen J, Ehrlich H, . Emergency resuscitative thoracotomy for civilian thoracic trauma in the field and emergency department settings: a systematic review and meta-analysis. J Surg Res. 2022;273:44-55. doi:10.1016/j.jss.2021.11.012 35026444

[37] Moore EE, Knudson MM, Burlew CC, ; WTA Study Group. Defining the limits of resuscitative emergency department thoracotomy: a contemporary Western Trauma Association perspective. J Trauma. 2011;70(2):334-339. doi:10.1097/TA.0b013e31829467c9 21307731

[38] Powell DW, Moore EE, Cothren CC, . Is emergency department resuscitative thoracotomy futile care for the critically injured patient requiring prehospital cardiopulmonary resuscitation? J Am Coll Surg. 2004;199(2):211-215. doi:10.1016/j.jamcollsurg.2004.04.004 15275875

[39] Tucker H, Ramage L, Greenhalgh R, . Trauma emergency thoracotomy for resuscitation in shock: a multi-centre evaluation of current UK practice of pre-hospital and emergency department resuscitative thoracotomy in trauma. J Surg Protoc Res Methodol. 2022;2022(4):snac011. doi:10.1093/jsprm/snac011

