# Supplementary material for: Prehospital Resuscitative Thoracotomy for Traumatic Cardiac Arrest
Source: JAMA Surg. 2025 Feb 26;160(4):432–40. doi: 10.1001/jamasurg.2024.7245 (PMC11866073; doi:10.1001/jamasurg.2024.7245)
Supplement: Supplement 1. — eTable 1. Surface location of injuries resulting in Traumatic Cardiac Arrest from Cardiac Tamponade and/or Exsanguination. eTable 2. Cardiac injuries resulting in Traumatic Cardiac Arrest from Cardiac Tamponade and/or Exsanguination. eTable 3. Survivors of traumatic cardiac arrest from cardiac tamponade. eTable 4. Survivors of traumatic cardiac arrest from exsanguination. eTable 5. Outcomes of 523 patients in traumatic cardiac arrest caused by either cardiac tamponade or exsanguination. eFigure. Number of (A) cases attended, (B) Traumatic Cardiac Arrest cases attended and (C) pre-hospital Resuscitative Thoracotomy procedures performed per year over the study period. [file jamasurg-e247245-s001.pdf]

Supplemental Online Content

Perkins ZB, Greenhalgh R, ter Avest E, et al. Prehospital resuscitative thoracotomy for traumatic cardiac arrest. *JAMA Surg*. Published online February 26, 2025. doi:10.1001/jamasurg.2024.7245

eTable 1. Surface location of injuries resulting in Traumatic Cardiac Arrest from Cardiac Tamponade and/or Exsanguination.
eTable 2. Cardiac injuries resulting in Traumatic Cardiac Arrest from Cardiac Tamponade and/or Exsanguination.
eTable 3. Survivors of traumatic cardiac arrest from cardiac tamponade.
eTable 4. Survivors of traumatic cardiac arrest from exsanguination.
eTable 5. Outcomes of 523 patients in traumatic cardiac arrest caused by either cardiac tamponade or exsanguination.
eFigure. Number of (A) cases attended, (B) Traumatic Cardiac Arrest cases attended and (C) pre-hospital Resuscitative Thoracotomy procedures performed per year over the study period.

This supplemental material has been provided by the authors to give readers additional information about their work.

© 2025 Perkins ZB et al. *JAMA Surgery*.

eTable 1: Surface location of injuries resulting in Traumatic Cardiac Arrest from Cardiac Tamponade and/or Exsanguination.

	Cardiac Tamponade (n = 105)		Cardiac Tamponade & Exsanguination (n = 72)		Exsanguination (n = 418)[a]	
	**Overall**	**Survivor**	**Overall**	**Survivor**	**Overall**	**Survivor**
**Cardiac Tamponade**						
Surface location of injury [b]						
Junctional (root of neck)	0	0	1 (1.4)	0	-	-
Thorax	96 (91.4)	21 (21.9)	67 (93.1)	0	-	-
Epigastrium	10 (9.5)	1 (10.0)	4 (5.6)	0	-	-
Abdomen	0	0	1 (1.4)	0	-	-
**Exsanguination**						
Site of Exsanguination						
Head/Face	-	-	0	0	2 (0.5)	0
Neck	-	-	0	0	18 (4.3)	0
Junctional (root of neck)	-	-	1 (1.4)	0	10 (2.4)	1 (10.0)
Junctional (Axilla)	-	-	0	0	2 (0.5)	0
Junctional (groin)	-	-	0	0	14 (3.4)	1 (7.1)
Thorax	-	-	72 (100)	0	270 (65.0)	3 (1.1)
Abdomen	-	-	3 (4.2)	0	94 (22.5)	2 (2.1)
Pelvis	-	-	0	0	24 (5.7)	0
Extremity (upper limb)	-	-	0	0	3 (0.7)	0
Extremity (lower limb)	-	-	0	0	21 (5.0)	1 (4.8)
Unclear	-	-	0	0	4 (1.0)	0
Single site of exsanguination	-	-	38 (52.8)	0	335 (80.1)	6 (1.8)
Multiple sites of exsanguination	-	-	34 (47.2)	0	83 (19.9)	2 (2.4)

Data presented as number (percent). Percentages may not add up to 100% as some patients had multiple injuries. [a] Cardiac injury relates to 194 patients that exsanguinated from cardiac or thoracic great vessel injuries. [b] Surface location of the injury that caused cardiac tamponade. [c] Data presented in the exsanguination group relates to any thoracic great vessel injury.

© 2025 Perkins ZB et al. *JAMA Surgery*.

eTable 2: Cardiac injuries resulting in Traumatic Cardiac Arrest from Cardiac Tamponade and/or Exsanguination.

	**Cardiac Tamponade** (n = 105)		**Cardiac Tamponade & Exsanguination** (n = 72)		**Exsanguination** (n = 418)[a]	
	**Overall**	**Survivor**	**Overall**	**Survivor**	**Overall**	**Survivor**
**Location of cardiac injury**						
Right Atrium	12 (11.4)	2 (16.7)	14 (19.4)	0	10 (5.2)	0
Left Atrium	1 (1.0)	0	8 (11.1)	0	2 (1.0)	0
Right Ventricle	64 (61.0)	14 (21.9)	29 (40.3)	0	52 (26.8)	0
Left Ventricle	28 (26.7)	4 (14.3)	20 (27.8)	0	64 (33.0)	0
Intrapericardial Great Vessel [c]	14 (13.3)	2 (14.3)	27 (37.5)	0	108 (55.7)	2 (1.9)
Unclear	3 (2.9)	0	2 (2.8)	0	0	0
Single cardiac injury	79 (75.2)	20 (25.3)	47 (65.3)	0	138 (71.1)	2 (1.5)
Multiple cardiac Injuries	26 (24.8)	2 (7.7)	25 (34.7)	0	56 (28.9)	0

Data presented as number (percent). Percentages may not add up to 100% as some patients had multiple injuries. [a] Cardiac injury relates to 194 patients that exsanguinated from cardiac or thoracic great vessel injuries. [b] Surface location of the injury that caused cardiac tamponade. [c] Data presented in the exsanguination group relates to any thoracic great vessel injury.

© 2025 Perkins ZB et al. *JAMA Surgery*.

eTable 3: Survivors of traumatic cardiac arrest from cardiac tamponade.

Survivor	Age/ Gender	MOI	Location of Cardiac Injury	Cardiac rhythm before intervention	TCA duration	Witnessed TCA? [a]	Aortic Occlusion	Internal Massage	Crystalloid (mls)	Blood (units)	Neurological outcome
1	27 M	Pent - Stab	LV	Sinus Tachycardia	≤ 1 min	Yes	NR	No	-	2	Good
2	29 M	Pent - Stab	RV	Sinus Tachycardia	≤ 1 min	Yes	Yes	No	100	4	Good
3	30 F	Pent - Stab	RV	Asystole	8 min	No	Yes	Yes	-	4	Poor
4	16 M	Pent - Stab	RV	Sinus Tachycardia	≤ 1 min	Yes	Yes	Yes	400	2	Good
5	46 M	Pent - Stab	RV	Sinus Tachycardia	≤ 1 min	Yes	Yes	No	300	-	Good
6	45 M	Pent - Stab	RV	Sinus Tachycardia	≤ 1 min	Yes	No	No	1500	-	Good
7	17 M	Pent - Stab	RV	Asystole	10 min	No	Yes	Yes	-	2	Poor
8	20 M	Pent - Stab	LV	Sinus Bradycardia	4 min	No	Yes	Yes	-	4	Good
9	22 M	Pent - Stab	LV	Sinus Tachycardia	≤ 1 min	Yes	No	No	2500	-	Good
10	25 M	Pent - Stab	RV	Asystole	10 min	No	Yes	Yes	500	-	Poor
11	33 M	Blunt - RTC	RA	Sinus Tachycardia	≤ 1 min	Yes	NR	No	3000	-	Good
12	16 M	Pent - Stab	NR	Sinus Tachycardia	≤ 1 min	Yes	No	No	50	-	Good
13	17 M	Pent - Stab	RV	Agonal	2 min	No	NR	Yes	1100	-	Good
14	48 M	Pent - Stab	RV	Asystole	15 min	No	NR	Yes	2500	-	Poor
15	18 M	Pent - Stab	RV	Agonal	5 min	No	NR	Yes	2000	-	Poor
16	41 M	Pent - Stab	RV	Sinus Tachycardia	≤ 1 min	Yes	Yes	Yes	600	-	Good
17	21 M	Pent - Stab	RV	Asystole	10 min	No	NR	Yes	1000	2	Good
18	16 M	Pent - Stab	RA	Sinus Bradycardia	≤ 1 min	No	Yes	NR	-	4	Good

© 2025 Perkins ZB et al. *JAMA Surgery*.

19	40 M	Pent - Stab	Great Vessel	Sinus Tachycardia	≤ 1 min	Yes	NR	Yes	-	2	Good
20	15 M	Pent - Stab	Great Vessel	Sinus Bradycardia	≤ 1 min	Yes	Yes	Yes	1500	-	Good
21	19 M	Pent - Stab	RV	Not recorded	3 min	No	NR	Yes	500	-	Good
22	18 M	Pent - Stab	RV	Sinus Bradycardia	3 min	No	Yes	Yes	1500	-	Poor

M, Male; Pent, Penetrating Injury; RV, Right Ventricle; LV Left Ventricle; RA, Right Atrium; min, minutes; NR, Not Recorded. [a] TCA witnessed by the advanced trauma team.

© 2025 Perkins ZB et al. *JAMA Surgery*.

eTable 4: Survivors of traumatic cardiac arrest from exsanguination.

Survivor	Age/ Gender	MOI	Site of Haemorrhage	Cardiac rhythm before intervention	TCA duration	Witnessed TCA? [a]	Procedure	Aortic Occlusion	Internal Massage	Crystalloid (mls)	Blood (units)	Neurological outcome
1	56 M	Pent - Stab	Abdomen	Sinus Tachycardia	≤ 1 min	Yes	Clamshell	Yes	Yes	2225	-	Good
2	52 M	Pent - Stab	Abdomen	Sinus Tachycardia	≤ 1 min	Yes	Clamshell	Yes	No	1500	-	Good
3	19 F	Pent - Stab	Thorax	Sinus Tachycardia	≤ 1 min	Yes	Clamshell	Yes	No	3000	-	Good
4	16 M	Pent - Stab	Root of neck	Sinus Tachycardia	5 min	No	Clamshell	Yes	Yes	1000	4	Good
5	29 M	Pent - GSW	Abdomen	Sinus Tachycardia	≤ 1 min	Yes	L Lateral	Yes	No	-	2 (WB)	Good
6	18 M	Pent - Stab	Thorax	Sinus Tachycardia	≤ 1 min	Yes	Clamshell	Yes	No	-	2 (WB)	Good
7	21 M	Pent - Stab	Thigh	Sinus Bradycardia	≤ 1 min	Yes	Clamshell	Yes	Yes	-	4	Poor
8	39 M	Pent - Stab	Thigh	Sinus Bradycardia	5 min	No	Clamshell	Yes	Yes	1500	3 (WB)	Good

M, Male; F, Female; Pent, Penetrating injury; min, minutes; WB, Whole Blood. [a] TCA witnessed by the advanced trauma team.

© 2025 Perkins ZB et al. *JAMA Surgery*.

eTable 5: Outcomes of 523 patients in traumatic cardiac arrest caused by either cardiac tamponade or exsanguination.

	Cause of Traumatic Cardiac Arrest			
	Cardiac Tamponade	Exsanguination	Unadjusted OR	P - Value
Outcome	(n = 105)	(n = 418)	(95% CI)	
Survival to Hospital Discharge	22 (21.0%)	8 (1.9%)	13.6 (6.0 to 29.5)	< 0.0001
Died on scene	46 (43.8%)	328 (78.5%)	0.2 (0.1 to 0.3)	< 0.0001
Survived Event [a]	59 (56.2%)	90 (21.5%)	4.7 (3.0 to 7.4)	< 0.0001
Survival with favourable neurological outcome [b]	16 (15.2%)	7 (1.7%)	10.6 (4.3 to 27.3)	< 0.0001

Data presented as number (percent). [a] Return of spontaneous circulation sustained until arrival at hospital and transfer of care to medical staff at the receiving hospital, [b] Cerebral Performance Category (CPC) Score of 1 or 2.

© 2025 Perkins ZB et al. *JAMA Surgery*.

eFigure. Number of (A) Cases attended, (B) Traumatic Cardiac Arrest cases attended, and (C) pre-hospital Resuscitative Thoracotomy procedures performed per year over the study period.

A

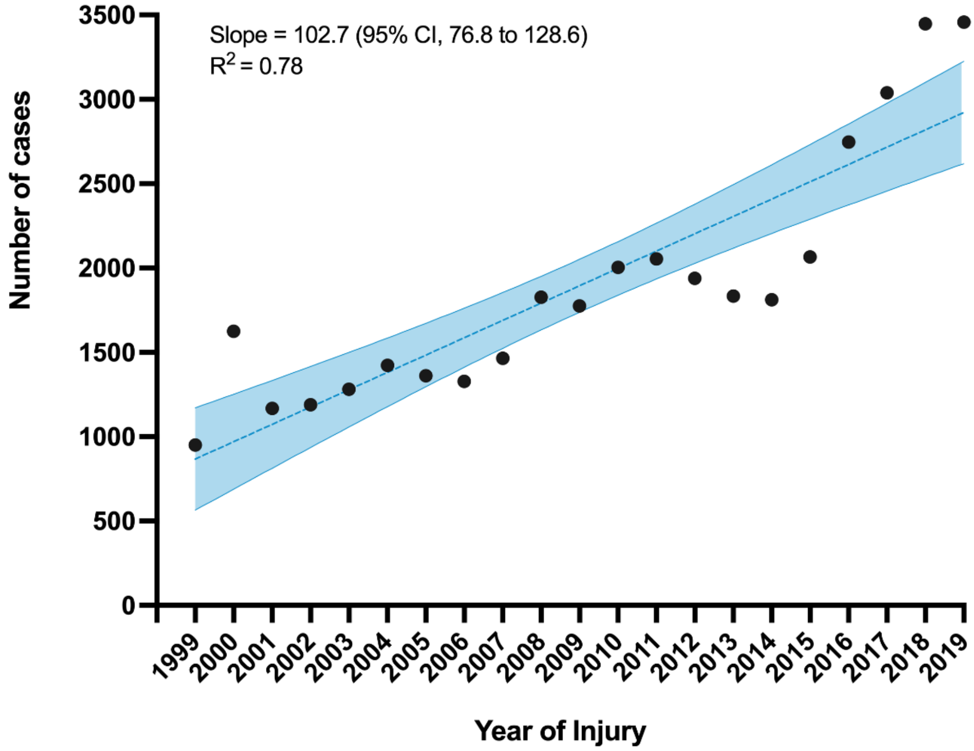

B

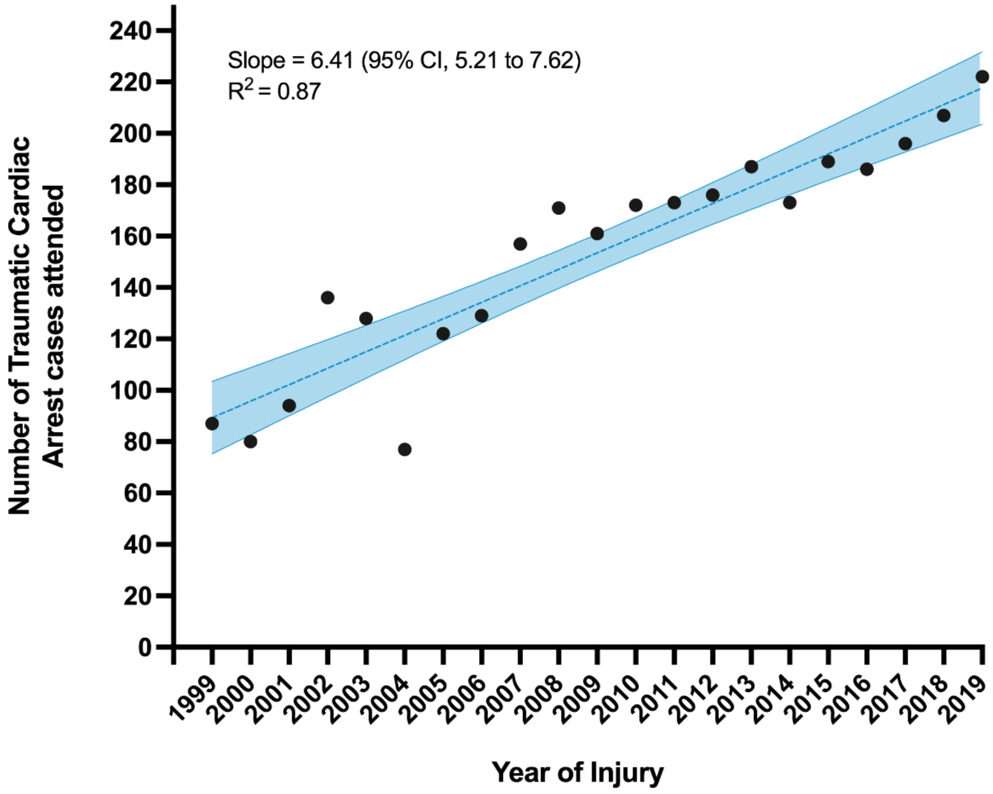

© 2025 Perkins ZB et al. *JAMA Surgery*.

C

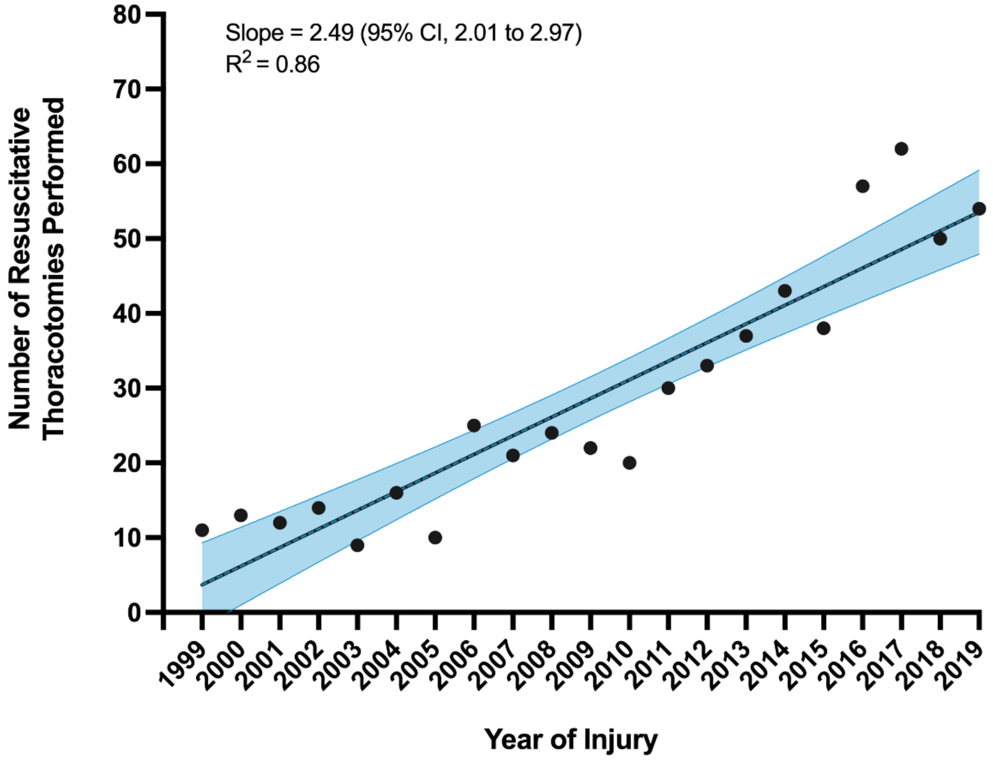

© 2025 Perkins ZB et al. *JAMA Surgery*.
