# Supplementary material for: Prehospital Resuscitative Thoracotomy for Traumatic Cardiac Arrest
Source: JAMA Surg. 2025 Feb 26;160(4):432–40. doi: 10.1001/jamasurg.2024.7245 (PMC11866073; doi:10.1001/jamasurg.2024.7245)
Supplement: Supplement 2. — Data sharing statement [file jamasurg-e247245-s002.pdf]

Data Sharing Statement

Perkins. Prehospital Resuscitative Thoracotomy for Traumatic Cardiac Arrest. *JAMA Surg*. Published February 26, 2025. doi:10.1001/jamasurg.2024.7245

Data
Data available: Yes
Data types: Deidentified participant data, Data dictionary
How to access data: The data collected and analyzed during the course of this observational cohort study are available upon reasonable request. All requests for data access will be considered by the corresponding author and the research team, pending approval from the institutional review board. The data will be shared in a de-identified format. For inquiries regarding data access, please contact (Dr Zane B. Perkins, z.perkins@qmul.ac.uk).
When available: With publication

Supporting Documents
Document types: None

Additional Information
Who can access the data: All requests for data access will be considered by the corresponding author and the research team, pending approval from the institutional review board.
Types of analyses: The data collected and analyzed during the course of this observational cohort study are available upon reasonable request.
Mechanisms of data availability: After approval of a proposal.
